# Identification of immunogenic cell death-related genes involved in Alzheimer’s disease

**DOI:** 10.1038/s41598-024-54357-6

**Published:** 2024-02-15

**Authors:** Rui Wang, Yaming Du, Wei Shao, Junli Wang, Xin Liu, Xinzi Xu, Guohua Chen, Yixuan Sun

**Affiliations:** 1 Hubei University of Chinese Medicine, 16 Huangjiahu West Road, Hongshan District, Wuhan, 430065 China; 2 Wuhan No. 1 Hospital, 215 Zhongshan Avenue, Qiaokou District, Wuhan, 430022 Hubei China; 3 The First Affiliated Hospital of Zhejiang Chinese Medical University (Zhejiang Provincial Hospital of Chinese Medicine), 54 Youdian Road, Shangcheng District, Hangzhou, 310003 Zhejiang China

**Keywords:** Alzheimer’s disease, Immunogenic cell death, WGCNA, GEO, Computational biology and bioinformatics, Neuroscience, Diseases, Neurology

## Abstract

Alzheimer's disease (AD) is the leading cause of dementia worldwide, with recent studies highlighting the potential role of immunogenic cell death (ICD) in the pathogenesis of this neurodegenerative disorder. A total of 52 healthy controls and 64 patients with AD were included. Compared to the controls, the patients with AD exhibited 2392 differentially expressed genes (DEGs), of which 1015 and 1377 were upregulated and downregulated genes, respectively. Among them, nine common genes were identified by intersecting the AD-related module genes with the DEGs and ICD-associated genes. Gene ontology (GO)analysis further revealed “positive regulation of cytokine production” as the most significant term. Moreover, the enriched molecular functions were primarily related to the inflammatory body complex, while the overlapping genes were significantly enriched in lipopolysaccharide binding. Kyoto encyclopedia of genes and genomes (KEGG) analysis also indicated that these overlapping genes were mainly enriched in immunity, inflammation, and lipid metabolism pathways. Furthermore, the following four hub genes were detected using machine learning algorithms: P2RX7, HSP90AA1, NT5E, and NLRP3. These genes demonstrated significant differences in expression between the AD and healthy control groups (P < 0.05). Additionally, the area under the curve values of these four genes were all > 0.7, indicating their potential diagnostic value for AD. We further validated the protein levels of these four genes in the hippocampus of 3xTg-AD and C57BL/6J mice, showing P2RX7 and HSP90AA1 expression levels consistent with the previously analyzed trends. Finally, the single-sample gene set enrichment analysis (ssGSEA) algorithm provided additional evidence by demonstrating the crucial role of immune cell infiltration and its link with the hub genes in AD progression. Our study results suggest that ICD-mediated elevation of HSP90AA1 and P2RX7 levels and the resulting induction of tau hyperphosphorylation and neuroinflammation are vital in the AD pathogenic mechanism.

## Introduction

Alzheimer’s disease (AD) is a chronic neurodegenerative disorder characterized by a gradual onset and progressive deterioration. This disorder primarily manifests as memory impairment, language difficulties, and cognitive decline, ultimately affecting the daily functioning of individuals with AD. These symptoms impose a remarkable burden on patients as well as lead to a considerable strain on the affected patients' families and society^1^. Consequently, AD has emerged as a critical global public health issue. According to the 2019 World Alzheimer’s Disease Report, the global prevalence of AD has reached 50 million, with the number projected to rise to 152 million by 2050. Moreover, AD has become the fifth leading cause of death worldwide^2^.

Despite extensive research, the underlying mechanisms involved in AD development remain elusive. Nevertheless, multiple theories have been proposed to explain AD onset, including the hypotheses concerning amyloid-beta (Aβ), tau protein hyperphosphorylation, gene mutation, neuroimmune response, cholinergic, synaptic dysfunction, and oxidative stress. These theories provide valuable insights into the complex nature of AD; however, further investigation is required to obtain a comprehensive understanding of this neurodegenerative disorder^3^. Furthermore, AD pathogenesis is multifactorial and involves complex mechanisms, with no specific drug treatment currently available in clinical practice. Neuroimmunity is increasingly considered pivotal in AD development^4–6^. Therefore, examining the role of immunity in AD has significant practical importance in advancing our understanding of AD and identifying potential strategies to alleviate this debilitating disease.

Immunogenic cell death (ICD) is a specific type of cell death that triggers the release of “danger signals,” such as disease-related or specific antigens, that can induce the immune system to mount an immune response. ICD possesses the characteristic ability to elicit an immune reaction and is a vital process in various pathological conditions^7,8^. Additionally, ICD is specifically associated with the release and increased expression of damage-associated molecular patterns (DAMPs), such as calreticulin, adenosine triphosphate (ATP), heat shock proteins (HSPs), and high mobility group box 1 (HMGB1) protein. These DAMPs act as signaling molecules, which activate and recruit antigen-presenting cells (APCs) and stimulate T cells to generate adaptive immune responses against disease antigens. Moreover, the binding of DAMPs to pattern recognition receptors (PRRs) initiates a series of immune events, ultimately triggering inflammation and immune responses within the local microenvironment. This interplay between DAMPs and PRRs is vital in regulating immune events and disease progression^9^. The concept of ICD initially emerged in cancer research, where the tumor cell death induced by certain anticancer drugs facilitated enhanced antitumor immune responses. Subsequent studies on the underlying mechanisms of ICD identified DAMPs as a critical component of ICD. Furthermore, research on ICD and DAMPs has expanded beyond cancer therapy, revealing its potential implications in non-infectious and non-tumor diseases. All these findings suggest that ICD may have broader therapeutic applications beyond its original context in cancer, offering new avenues for exploring the immunomodulatory effects of cell death in various pathological conditions^10^. The association of ICD and endogenous DAMPs to some extent in non-neoplastic diseases is gaining traction^11^. Numerous studies have demonstrated the involvement of DAMPs in neuroinflammation in various neurodegenerative diseases, including AD, the most prevalent neurodegenerative disorder. These DAMPs predominantly originate intracellularly, from where they are released by different cellular compartments or organelles in response to tissue stress or cell death. Furthermore, DAMPs contribute to innate immune activation in AD, thereby fostering an oxidative and neuroinflammatory milieu within the affected brain^12^. Moreover, previous observations indicate that individuals with AD exhibit three critical conditions for ICD occurrence: antigenicity, adjuvant effects, and microenvironmental factors. First, neuronal cell death leads to the extracellular release of a substantial amount of amyloid-beta (Aβ), eliciting an immune response and conferring antigenicity^13^. Second, DAMPs (such as HSPs and HMGB1 proteins) released during ICD are closely associated with AD, functioning as adjuvants in the immune response^12^. Lastly, the neurons of individuals with AD present with several characteristics that resemble the ICD microenvironment and are conducive to ICD, including hypoxia^14^, pH reduction^15^, and inflammation^16^.

The application of next-generation sequencing technologies has enabled vast amounts of high-quality human sample sequences to be submitted and stored in public databases (such as the Gene Expression Omnibus [GEO]), which has facilitated data sharing among researchers globally^17^. This wealth of sequence information also provides a convenient and practical resource for integrating and analyzing the pathological mechanisms underlying AD. Moreover, weighted gene co-expression network analysis (WGCNA) has emerged as a novel systems biology approach to transform genes into co-expression modules and network signals, thereby unraveling the intricate relationships between genes and phenotypes^18^.

In this study, we aimed to explore the association between ICD and AD at the genetic level. For this purpose, we initially obtained the expression matrices and corresponding clinical information from two datasets in the GEO database. Gene re-annotation was then performed on the probe sets of the datasets, comprising healthy control and AD brain tissue samples. The expression information from the datasets was merged, and batch effects were eliminated. Next, differentially expressed genes (DEGs) between the AD and healthy control samples were identified using differential gene expression analysis and WGCNA. Subsequently, the genes associated with ICD were determined by intersecting with the DEGs. Further, gene ontology (GO) enrichment and Kyoto Encyclopedia of Genes and Genomes (KEGG) pathway analyses were conducted to elucidate the potential functions of the key gene modules. LASSO analysis and random forest machine learning methods were also employed to screen for the intersection genes, and an optimal hub gene was identified. The accuracy of the hub gene for AD diagnosis was then assessed using receiver operating characteristic (ROC) curve analysis. Additionally, single-sample gene set enrichment analysis (ssGSEA) was used to investigate the differences in the immune infiltration ratio and immune pathway activity between the healthy control and AD groups. Moreover, the correlation of the expression level of the hub gene with immune cell infiltration ratio and immune pathway activity in the AD group was evaluated. Furthermore, the identified hub genes were validated using AD animal models. Therefore, the final selected hub gene may serve as an ICD-related biomarker for AD diagnosis and treatment monitoring and provide insights into ICD as a potential therapeutic target for AD.

## Materials and methods

### Data acquisition and processing

Microarray expression data for AD and its clinical information were obtained from the GEO database (http://www.ncbi.nlm.nih.gov/geo/): GSE122063 (https://www.ncbi.nlm.nih.gov/geo/query/acc.cgi?acc=GSE122063) and GSE37263 datasets (https://www.ncbi.nlm.nih.gov/geo/query/acc.cgi?acc=GSE37263). The database probe was gene annotated. A total of 136 brain tissue samples were included from the GSE122063 dataset, consisting of 44 and 56 samples from healthy controls and AD, respectively. Additionally, forty brain tissue samples were acquired from the GSE37263 dataset, with eight samples each from the AD and healthy controls. Finally, using the “sva” R package and based on the batch effect, the expression information of the two datasets was merged, and the between-batch differences were eliminated.

### Identification of DEGs

Differential gene expression analysis between the AD and healthy control groups was performed on the data of the GSE122063 and GSE37263 datasets using the “limma” R package, with DEG screening criteria consisting of adjusted P-value < 0.05 and |log fold change (FC)| > 0.5.

### Weighted gene co-expression network analysis

The gene co-expression network was constructed by implementing the WGCNA R package^19^, while the gene co-expression matrix was generated using Pearson’s correlation analysis. Subsequently, the soft threshold value was selected to build the scale-free network according to the scale-free network principle. After estimating the threshold value, the adjacency matrix was transformed into a topological overlap matrix. Cluster analysis was then conducted to identify the gene modules, in which the minimum number of genes in each module was set to 60. Furthermore, a tree was constructed via hierarchical clustering to calculate the correlation between the characteristic genes in the module and the disease phenotype. This process helped to screen the distinct gene groups of the module, and the module with the largest correlation coefficient and the smallest P value was selected as the module representing the disease-related characteristics. Finally, the hub gene of the module was extracted. In this step, the screening conditions for the hub gene were a gene importance score of > 0.4 and a correlation of > 0.5 between the gene and module.

### Intersection of Alzheimer’s disease-related module genes with differentially expressed genes and immunogenic cell death-related genes

The ICD-associated genes were obtained from previously published literature^20,21^. We overlapped the DEGs and ICD-related genes with AD-associated module genes derived from WGCNA. Furthermore, a Venn diagram was used to visualize the details of the overlapping genes.

### Functional enrichment analysis of overlapped genes

Functional enrichment analysis was performed in the three domains of GO, i.e., biological process (BP), cellular component (CC), and molecular function (MF). Moreover, the KEGG database contains the datasets of pathways involving biological functions, diseases, and chemicals and drugs^22^. The enrichment analysis was conducted using the “limma,” “org.Hs.eg.db,” “clusterProfiler,” and “enrichplot” R packages to determine the biological functions of the genes and associated pathways^23^.

### Screening of hub genes

The candidate hub genes were screened using the “glmnet” R package to perform LASSO analysis^24^. Additionally, the random forest machine learning method was employed to compress the intersection genes and detect the optimal variables, followed by taking the intersection of the genes obtained by the two machine learning methods.

### Differential expression analysis and receiver operating characteristic curve validation

The R limma packages were used to evaluate and compare the expression levels of the final hub genes in the AD group with those in the control group and display this data via boxplots^25^. Moreover, ROC curve analysis was performed for each hub gene using the R pROC package^26^, and the area under the curve (AUC) with a 95% confidence interval (CI) was calculated. The significance of ICD was based on the AUC, with values close to 1 indicating higher accuracy of the model training.

### Animals

All procedures concerning the experimental animals were performed in accordance with the relevant guidelines and regulations.

### Mice

Adult 3xTg-AD male mice (aged 6–7 weeks) were procured from Changzhou Cavens Laboratory Animal Co., Ltd. (Wuhan, China; license no.: SCXK (Su) 2016-0010). The mice were maintained in a specific pathogen-free laboratory of Wuhan Myhalic Biotechnology Co., Ltd. (Wuhan, China) at 20–26 °C, humidity of 50% ± 10%, and 12-h day and night light cycle. Mice were fed standard feed pellets and had free access to water and food up to 9 months of age. Adult C57BL/6J male mice (aged 7 months) were obtained from Wuhan Youdu Biotechnology Co., Ltd. (Wuhan, China; license no.: SCXK (E) 2021-0025). These mice were housed under the same conditions as the 3xTg-AD mice, including similar feeding up to 9 months of age. All animal studies were performed with the approval of the Animal Ethics Committee of Wuhan Myhalic Biotechnology Co., Ltd. (ethical approval number: HLK-20230518-001). All experimental procedures adhered to the ARRIVE guidelines and the American Veterinary Medical Association Guidelines for the Euthanasia of Animals (2020). In this study, the mice did not undergo any interventional experimental methods. The three adult 3xTg-AD and three C57BL/6J mice were anesthetized with isoflurane. After anesthesia, the mice were decapitated, and the hippocampal tissues were rapidly extracted for western blot experiments.

### Western blot analysis

Mice hippocampal tissues were taken and plasma nuclei were separated according to the nucleus protein extraction kit, protein concentration was determined by BCA method, boiling was performed for denaturation, and then the proteins were separated via SDS-PAGE and transferred onto nitrocellulose membranes. The membranes were then re-probed with an antibody specific to GAPDH (37 kDa) as an internal control. Additionally, HSP90AA1, NLRP3, NT5E, and P2RX7 were detected using anti-rabbit HSP90α (101 kDa), anti-rabbit NLRP3 (118 kDa), anti-rabbit NT5E (70 kDa), and anti-rabbit P2RX7 antibodies (75 kDa), followed by HRP-labeled goat-anti rabbit IgG and goat-anti mice IgG antibodies. Finally, the X-ray films were developed and fixed in a dark room.

### Assessment of immune cell infiltration and its correlation with hub genes

The “gsva” R package was used for ssGSEA enrichment analysis to investigate immune cell infiltration and its association with the hub genes^27^. Initially, the immune cell infiltration ratio of each sample was calculated based on the expression matrix of the AD group samples, and the difference in the immune cell infiltration ratio between the healthy control and AD groups was compared via the “vioplot” R package. Furthermore, the “pheatmap” R package was applied to visualize the immune cell infiltration between the two groups. Lastly, using the “ggplot2” and “reshape2” R packages and based on the Spearman rank correlation test, a correlation comparison of the expression level of the hub gene with the proportion of immune cell infiltration in the AD group was conducted.

### Assessment of immune function and its correlation with hub genes

The gsva R package was also applied for ssGSEA enrichment analysis to evaluate immune function and its correlation with the hub genes using the similar steps described for assessing immune cell infiltration. The enrichment score of the immune pathway of each sample was first estimated according to the expression matrix of the AD group samples. Next, the difference between the enrichment scores of the immune pathways of the healthy control and AD groups was compared via the R vioplot package, while the R pheatmap package was employed to visualize the immune functions between the two groups. Finally, after applying the R ggplot2 and reshape2 packages and based on the Spearman rank correlation test, the correlation between the expression level of the hub gene and the enrichment score of the immune pathway in the AD group was compared.

## Results

### Data acquisition and processing

**Figure 1:**
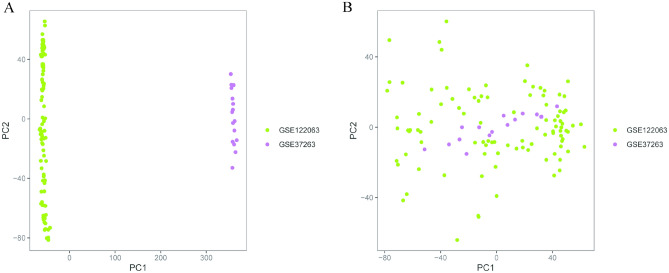
Principal component analysis (PCA) diagram. (**A**) Distribution of data dimensions before merging the two datasets and eliminating batch differences. (**B**) Distribution of data dimensions for the two datasets merged after eliminating batch differences.

The GSE37263 dataset contained eight brain tissue samples each of the healthy controls and patients with AD, while the GSE122063 dataset comprised 44 and 56 samples of the healthy controls and patients with AD, respectively. Eventually, 52 and 64 brain tissue samples of the healthy controls and patients with AD were obtained after eliminating batch differences and merging. Figure 1 illustrates the data dimensions before and after the consolidation of the two datasets.

### Identification of DEGs and screening of hub genes

**Figure 2:**
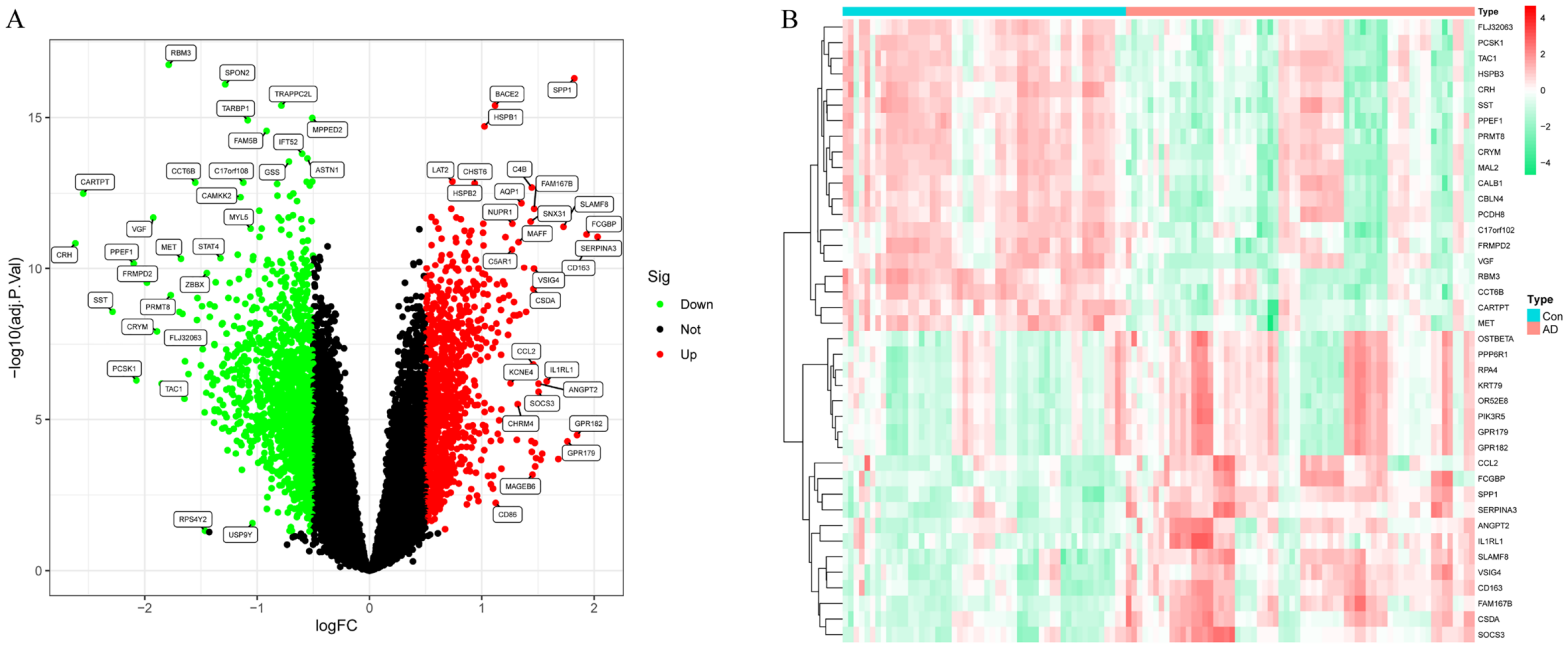
Identification of differentially expressed genes (DEGs) and screening of hub genes. (**A**) Volcano plot for DEGs between the healthy control and AD brain tissues. Red color represents differential genes up-regulated in AD samples and green color represents differential genes down-regulated in AD samples. (**B**) Heat map of the top 20 up-regulated and top 20 down-regulated differential genes.

A total of 2392 DEGs, including 1015 up-regulated and 1377 down-regulated genes, were obtained based on an adjusted P-value of < 0.05 and |logFC| of > 0.5. The DEGs are presented using volcano plots in Fig. 2A. A heat map displays the top 20 up-regulated and top 20 down-regulated differential genes in Fig. 2B.

### Weighted gene co-expression network construction and identification of core modules

**Figure 3:**
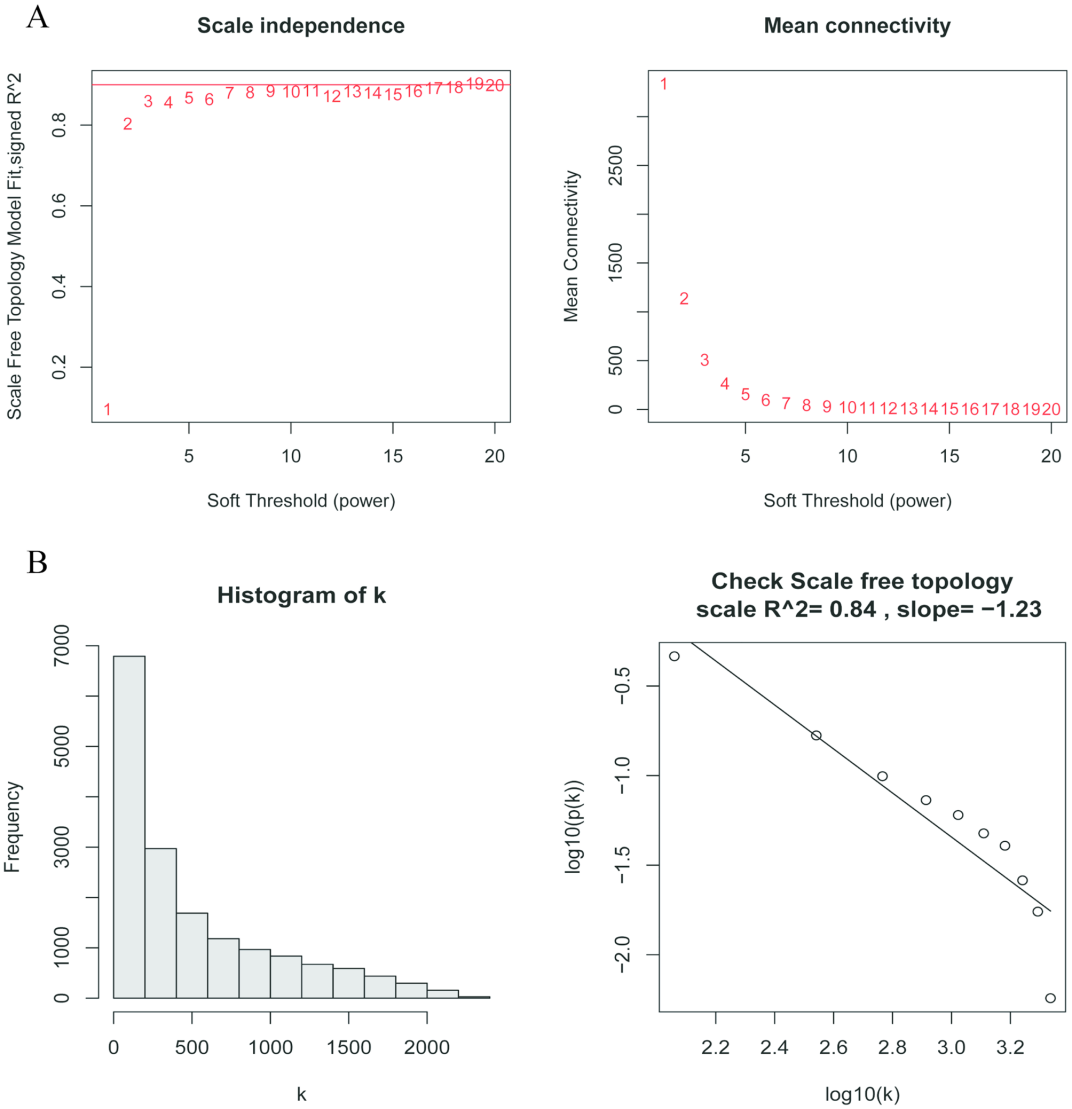
Determination of soft-thresholding power in the weighted gene co-expression network analysis (WGCNA). (**A**) Analysis of the scale-free fit index and the mean connectivity for the various soft-thresholding powers (β). The corresponding soft-thresholding power is 3. (**B**) Histogram of connectivity distribution and checking the scale-free topology.

**Figure 4:**
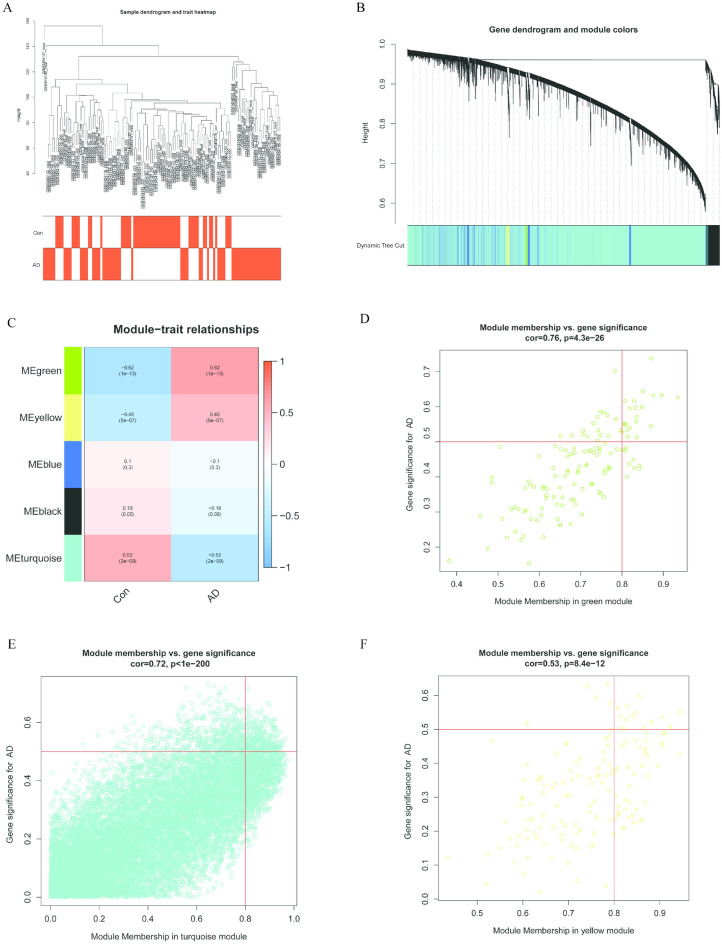
Construction of WGCNA modules. (**A**) Sample dendrogram and clinical grouping features based on color annotation. (**B**) Each branch represents one gene, with every color below denoting one co-expression module. (**C**) Heatmap of the module-trait relationships. The green, turquoise, and yellow modules are significantly associated with AD. (**D**–**F**) Scatter plot for the correlation between gene module membership in the green, turquoise, and yellow modules and gene significance.

The scale-free network was generated with the soft threshold set to 3 (R^2^ = 0.84) for consistency (Fig. 3A,B). The adjacency matrix was then created using a weighted correlation coefficient. Subsequently, the adjacency matrix was transformed into a topological overlap matrix. Hierarchical clustering was further performed to identify modules, and the eigengene was calculated. The genes in the green, yellow, and turquoise-colored modules were highly correlated, and the hub genes of these three modules were extracted (Fig. 4). The conditions for hub gene screening in the modules were a gene importance score of > 0.4 and a correlation of > 0.5 between the gene and the module in the most relevant module of the disease.

### Intersection of Alzheimer’s disease-related module genes with differentially expressed genes and immunogenic cell death-related genes and enrichment analysis

**Figure 5:**
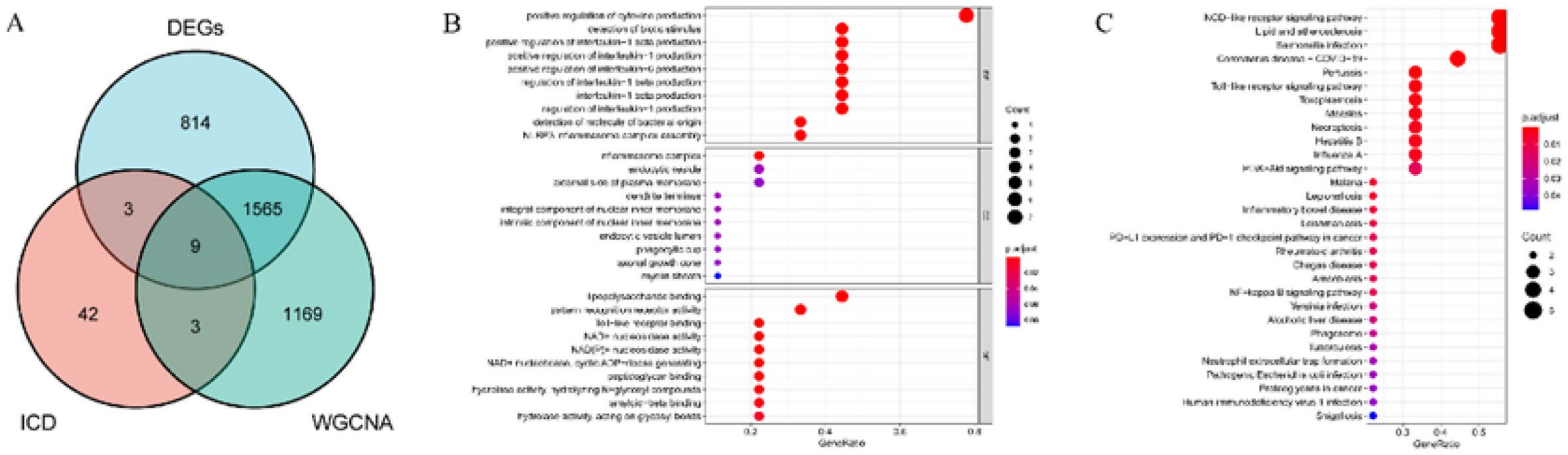
(**A**) A Venn diagram for the intersections between differentially expressed genes (DEGs), differential genes based on weighted gene co-expression network analysis (WGCNA), and immunogenic cell death (ICD)-related genes. (**B**) Gene ontology (GO) functional analysis showing the enrichment of the overlapped genes. (**C**) Kyoto Encyclopedia of Genes and Genomes (KEGG) pathway enrichment analysis of the overlapped genes.

A total of 57 ICD-related genes were identified from previous literature (Table S1). Eventually, nine intersecting genes were obtained based on the intersections of the DEGs, AD-related module genes (WGCNA), and ICD-related genes (Fig. 5A), including P2RX7, NLRP3, HSP90AA1, TLR2, LY96, TLR4, IFIH1, NT5E and AIM2. The significant GO functional terms of the nine overlapped genes in terms of the BP, MF, and CC domains are illustrated in Fig. 5B. In GO-BP, the intersection genes were principally associated with the significant term “positive regulation of cytokine production.” In the case of GO-MF, the principal term was “inflammasome complex.” Further, the GO-CC indicated that the overlapped genes were significantly enriched in the CC term “lipopolysaccharide binding.” Lastly, the KEGG analysis showed that these overlapped genes were enriched in the NOD-like receptor signaling pathway (Fig. 5C). The top five pathways enriched by KEGG were: NOD-like receptor signaling pathway, Toll-like receptor signaling pathway, PI3K-Akt signaling pathway, PD-L1 expression and PD-1 checkpoint pathway in cancer, NF-kappa B signaling pathway.

### Machine learning algorithms

**Figure 6:**
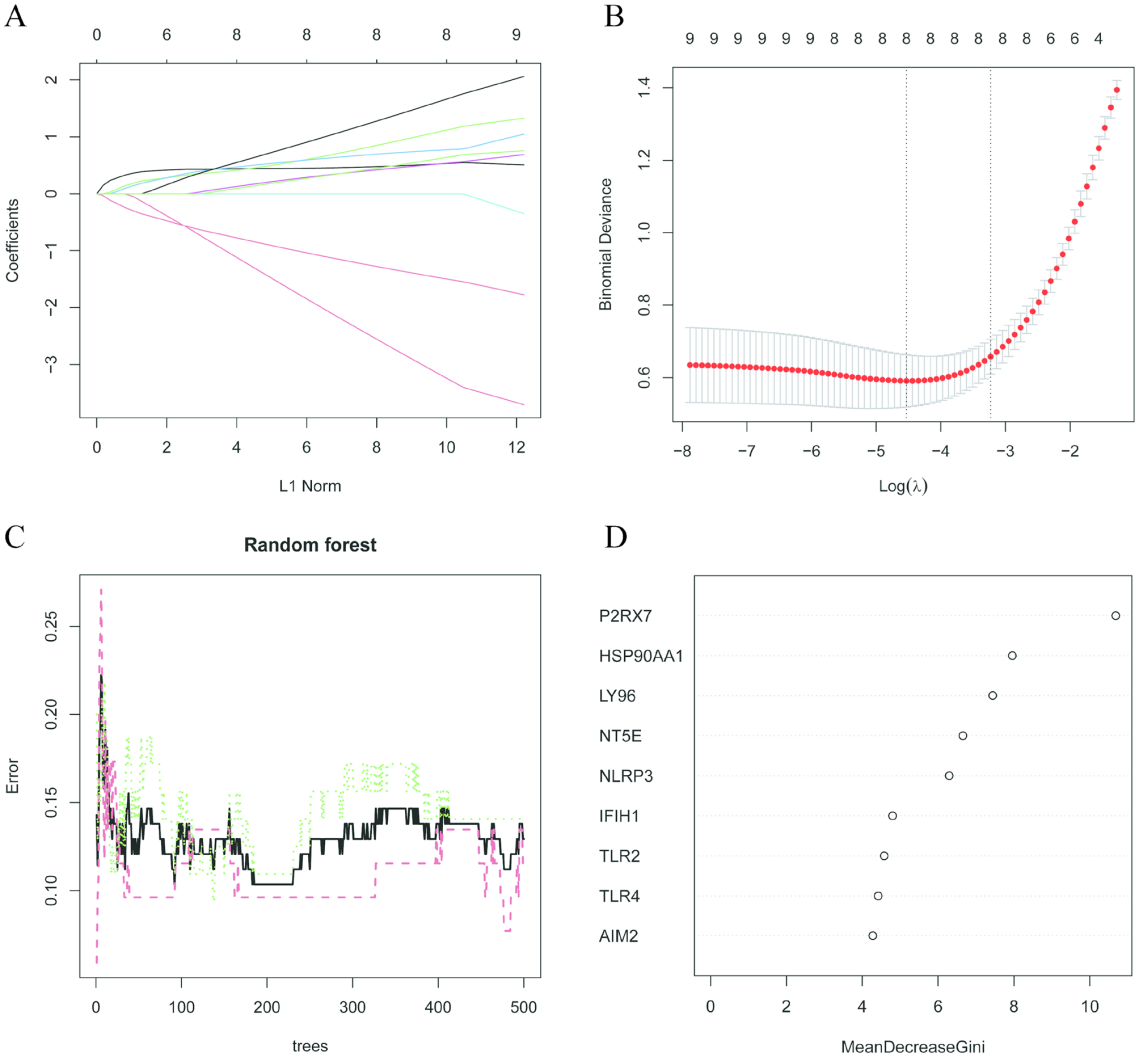
(**A**) Variable selection using LASSO binary logistic model. A coefficient profile plot is constructed against the L1 Norm sequence. (**B**) Eight variables with nonzero coefficients are selected by deriving the optimal lambda. After verifying the optimal parameter (lambda) in the LASSO model, the partial likelihood deviance (binomial deviance) curve versus log(lambda) is plotted, and dotted vertical lines are drawn based on 1 standard error criteria. (**C**) Random forest chart: The horizontal axis represents the number of trees, while the vertical axis denotes the error in the loss function. Smaller errors indicate a better model fit to the data. Moreover, selecting the optimal number of trees is essential to achieving the best model performance. (**D**) The variable importance is compared by calculating the influence of each variable on the heterogeneity of the observations at each node of the classification tree, wherein a higher value signifies greater variable importance.

LASSO (Least absolute shrinkage and selection operator) can eliminate the regression coefficients of some unnecessary variables from the model by adding a penalty term in the model estimation, so as to achieve the purpose of variable selection. In lasso analysis, the binomial type of lasso regression model was choosed; the biomarkers in this model were choosed by tenfold cross validation, which was used with cv.glmnet function. finally, the lambda.min was selected as the optimal lambda value and generated this lasso model. through the construction of the diagnostic model, we use the variables in the lasso model as biomarkers for our final screening. Initially, the regression yielded eight genes: P2RX7, NLRP3, HSP90AA1, TLR2, TLR4, IFIH1, NT5E, and AIM2 (Fig. 6A,B). Subsequently, the random forest machine learning method was applied to compress the nine intersection genes, and five genes, including P2RX7, HSP90AA1, NT5E, NLRP3, and LY96, were obtained (Fig. 6C,D). Finally, the intersection of the genes obtained by the two machine learning methods was utilized to acquire the final four hub genes: P2RX7, HSP90AA1, NT5E, and NLRP3.

### Differential expression analysis and receiver operating characteristic curve validation

**Figure 7:**
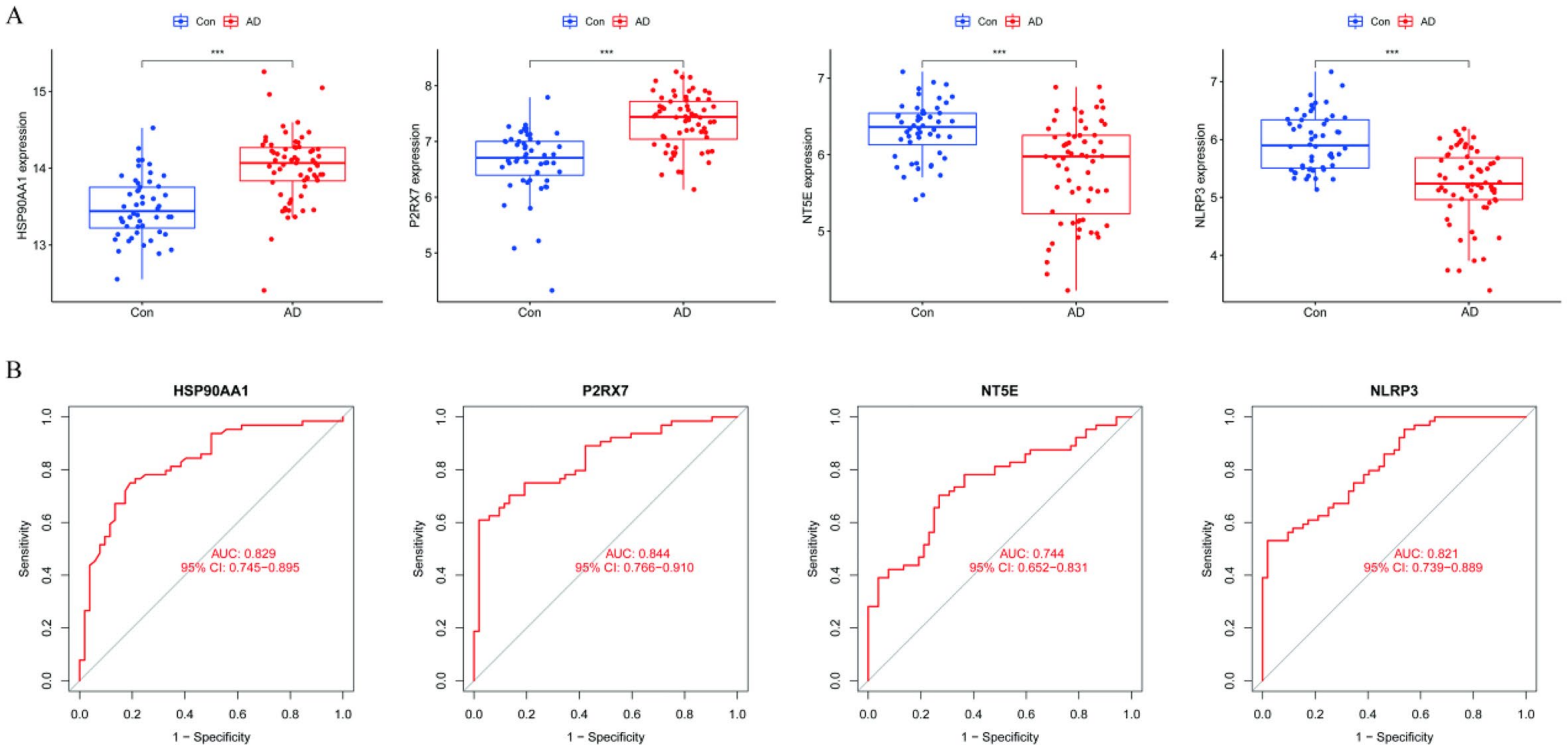
(**A**) Validation of the hub genes at the gene expression level. (**B**) Validation of the diagnostic value of the hub genes in AD.

The expression levels of the four hub genes were validated using box plots (Fig. 7A). The analysis demonstrated significantly higher expression levels of HSP90AA1 and P2RX7 (P < 0.001) and significantly lower expression levels of NLRP3 and NT5E (P < 0.001) in the brain tissues of patients with AD than in those of healthy controls. In the ROC curve analysis, the AUC values of the four hub genes were compared to assess their sensitivity and specificity for AD diagnosis. All four hub genes exhibited AUC values > 0.70, indicating their diagnostic value for this neurodegenerative disorder (Fig. 7B).

### Western blot analysis

**Figure 8:**
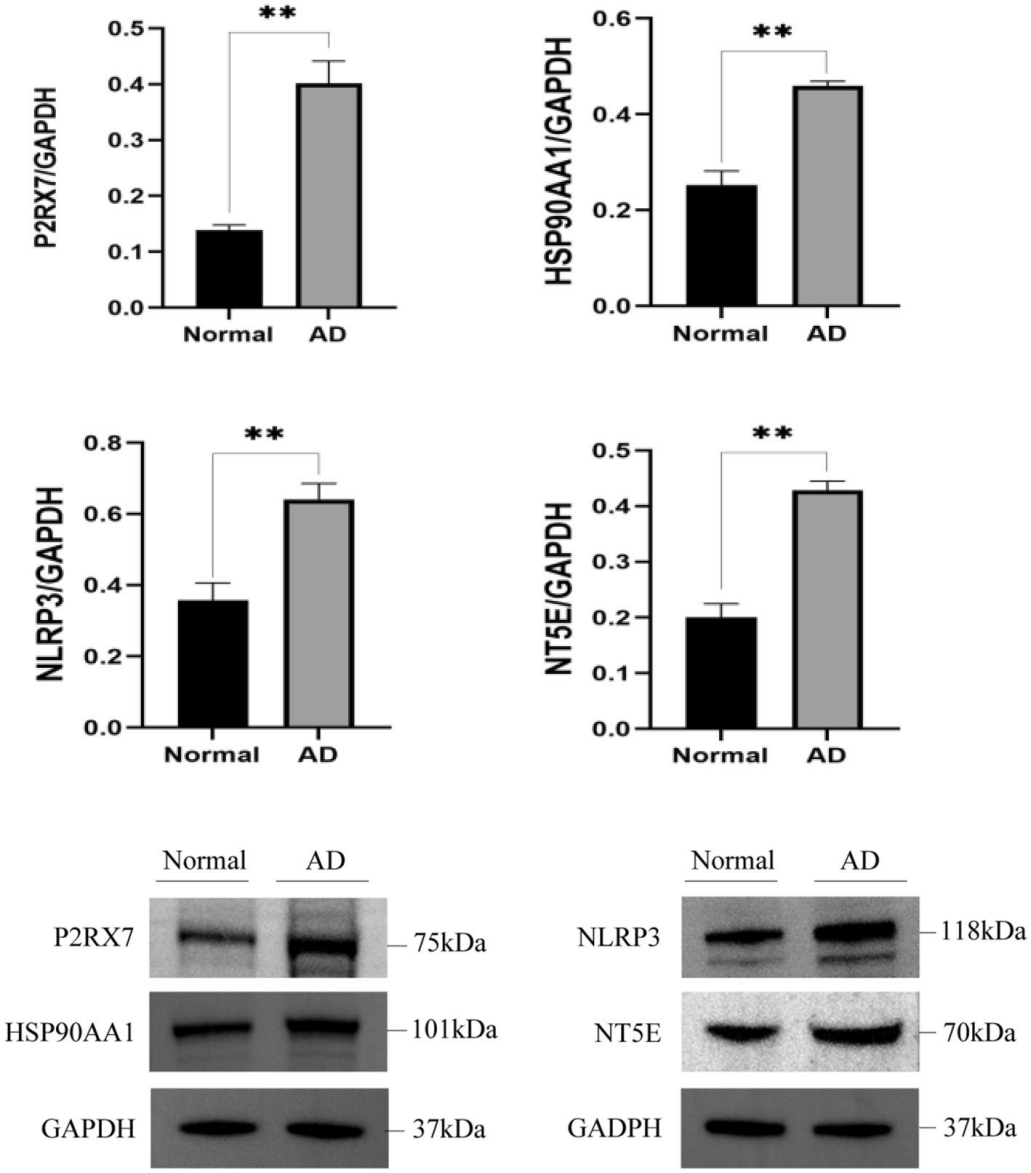
Expression of P2RX7, HSP90AA1, NLRP3, and NT5E in the hippocampus of normal and AD mice. **P < 0.01. The samples derive from the same experiment and that gels/blots were processed in parallel. Original blots/gels are presented in Supplementary Fig. 1.

Based on the previous analysis, HSP90AA1 (HSP90α), P2RX7, NT5E, and NLRP3 were selected for experimental verification based on western blot analysis. The results showed that the levels of these four proteins were significantly increased in the hippocampus of the AD mice group compared to the normal mice group (P < 0.01; Fig. 8).

### Immune cell infiltration and its correlation with hub genes

**Figure 9:**
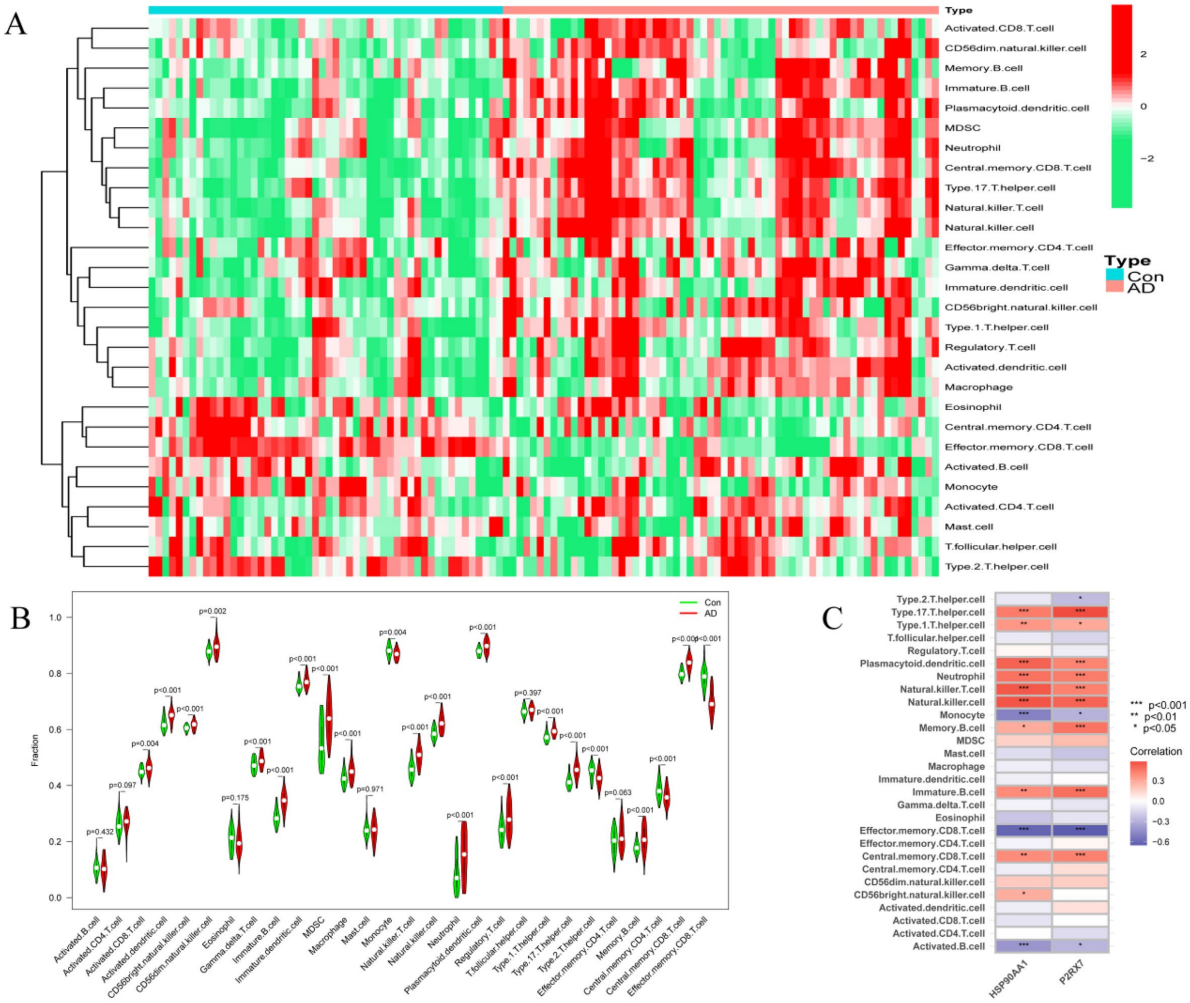
Analysis of the immune landscape associated with AD. (**A**) Heatmap and (**B**) violin plot showing the distribution of 28 types of immune cells in the healthy control and AD brain tissues. (**C**) The relationship between the two hub genes (HSP90AA1 and P2RX7) and immune cell infiltration.

To further investigate the differences in immune cell infiltration between the patients with AD and healthy controls, their relationship was assessed using the ssGSEA algorithm. The distribution of 28 immune cells in the GSE37263 and GSE122063 samples is depicted in Fig. 9A. The results of the immune cell infiltration analysis demonstrated a significantly higher infiltration of monocytes, CD4^+^ T cells, CD8^+^ T cells, regulatory T cells, and natural killer (NK) cells in the brain tissues of the patients with AD than in those of the healthy controls, suggesting that these cells were essential in AD progression (Fig. 9B). Correlation analysis of the 28 immune cells with the hub genes showed that type 17 and type 1 T helper cells, plasmacytoid dendritic cells, neutrophils, NK/T cells, NK cells, memory and immature B cells, and central memory CD8 T cells positively correlated with HSP90AA1 and P2RX7 (all P < 0.05). Conversely, monocytes, effector memory CD8 T cells, and activated B cells negatively correlated with HSP90AA1 and P2RX7 (all P < 0.05). Additionally, CD56^bright^ NK cells positively correlated with HSP90AA1, whereas type 2 T helper cells negatively correlated with P2RX7 (Fig. 9C). All these results further indicated the crucial role of these immune cells in AD progression.

### Immune function and its correlation with hub genes

**Figure 10:**
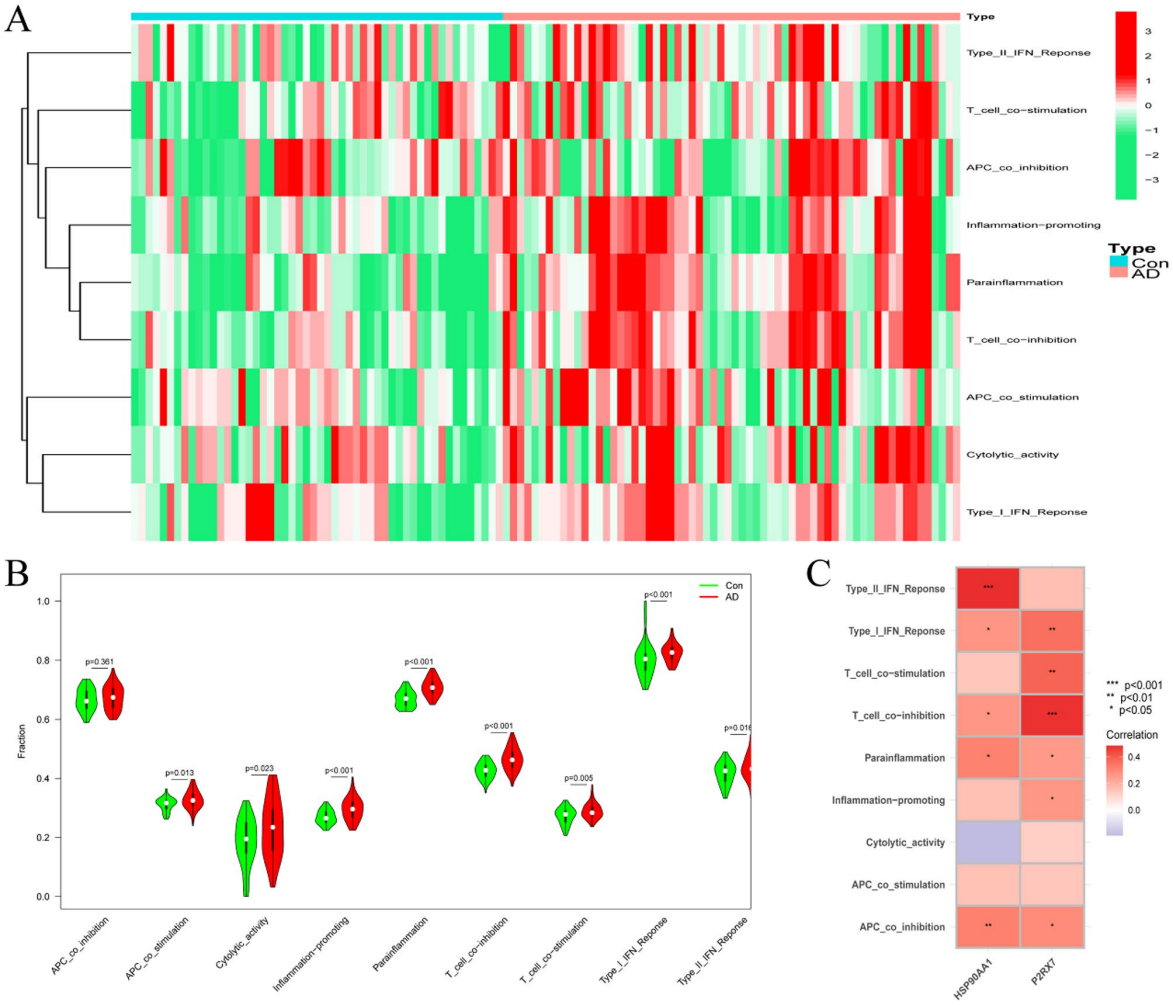
Analysis of the immune landscape linked to AD. (**A**) Heatmap and (**B**) Violin plot displaying the distribution of nine types of immune functions in the healthy control and AD brain tissues. (**C**) The relationship between the two hub genes (HSP90AA1 and P2RX7) and immune function.

ssGSEA (single sample gene set enrichment analysis), can be used to calculate enrichment scores for individual samples and gene set pairs, where each enrichment score represents the extent to which the genes in a particular gene set are coordinately up-regulated or down-regulated in a sample, enabling the characterisation of cellular traits based on the level of activity of characteristic biological processes and pathways. Similar to the immune cell infiltration investigation, the ssGSEA algorithm was also applied to examine the variation in the immune function between the patients with AD and healthy controls. The distribution of nine immune functions in the merged AD samples is presented in Fig. 10A. The findings of the immune function analysis exhibited significantly higher levels of APC co-stimulation, cytolytic activity, inflammation promotion, parainflammation, T cell co-inhibition, T cell co-stimulation, and type I and II IFN responses in the brain tissues of the patients with AD than in those of the healthy controls, implying that these functions were vital in AD progression (Fig. 10B). Correlation analysis of the nine immune functions with the hub genes indicated that type I IFN response, T cell co-inhibition, parainflammation, and APC co-inhibition positively correlated with HSP90AA1 and P2RX7 (all P < 0.05). Furthermore, type II IFN response positively correlated with HSP90AA1, while inflammation promotion positively correlated with P2RX7 (Fig. 10C). All these results suggest that these immune functions are pivotal in AD progression.

## Discussion

ICD, initially conceptualized in cancer research studies, enhances immune response by releasing antigens and adjuvants from dying cells^28^. During ICD, dying cells generate novel antigenic epitopes and release DAMPs. Further, these DAMPs recruit APCs, facilitate the recognition and phagocytosis of dead cell antigens, and present them to T cells, thereby activating the adaptive immune response. This adaptive immune response is vital in recognizing and clearing tumor antigens, ultimately leading to a durable antitumor effect^29^. Moreover, emerging evidence suggests that normal cells can trigger antigen-specific immune responses under specific insults such as oxidative stress and hyperglycemia, which in turn contribute to disease pathogenesis^30^. The DAMPs produced during ICD may also have prominent implications in non-malignant and non-infectious diseases^11,31,32^.

Although the precise association between ICD and AD requires further elucidation, our study sheds light on several pertinent observations. First, neuronal cell death induces the extracellular release of a substantial amount of neuronal Aβ, triggering an immune response and antigenic properties^13^. Second, ICD leads to the release of DAMPs (such as HSPs and HMGB1 proteins) that are closely linked to AD^12^ and potentially act as adjuvants during AD pathology. Lastly, neurons affected by AD exhibit characteristic brain microenvironment features, including hypoxia^14^, altered pH^15^, and an inflammatory response^16^, closely resembling the microenvironment conducive to ICD. With WGCNA analysis, a network can be constructed based on systematic gene expression levels, thus showing co-expression relationships between genes; these genes are assigned to different modules, then genes within the same module have similar expression patterns and may be co-regulated or functionally related. In addition, there are correlations between different modules and phenotypes. Therefore, by screening AD-related modules it helps to obtain the expression trends of certain genes that share the same trend along with the development of AD. Therefore, through WGCNA analysis, it can well reflect which genes are subsequently co-regulated or functionally interacted when AD occurs, and thus better illustrate the biological characteristics of AD by screening this group of genes. Based on these findings, this study employed the WGCNA method to analyze and identify ICD-related disease signature genes in patients with AD. Our study results may provide novel insights that aid in the early diagnosis of AD and inform targeted drug research for treating this neurodegenerative disorder.

In this study, we initially employed differential gene expression analysis and WGCNA to identify DEGs in AD. Subsequently, we intersected these genes with ICD-related genes and identified nine overlapping genes. Next, GO analysis revealed that these genes were primarily enriched in various biological processes relevant to AD pathogenesis, including immune response (e.g., PRR activity, toll-like receptor [TLR] binding, and peptidoglycan binding), inflammatory response (e.g., lipopolysaccharide binding), mitochondrial function (e.g., NAD^+^ nucleotidase, cyclic ADP-ribose generation, and NAD[P]^+^ nucleosidase activity), glucose metabolism (e.g., hydrolase activity, hydrolyzing N-glycosyl compounds, hydrolase activity, and acting on glycosyl bonds), and Aβ metabolism (e.g., Aβ binding)^33–36^. Furthermore, the analysis of the KEGG signaling pathways demonstrated that these genes were predominantly enriched in pathways linked to immune and inflammation-related diseases (e.g., NOD-like receptor, TLR, and NF-kappa B signaling pathways), as well as glucose and lipid metabolism (e.g., lipid and atherosclerosis pathway and PI3K-Akt signaling pathway). In particular, NLRP3, a member of the NLR family, was found to be crucial in AD pathogenesis, with NLRP3 activation being linked to the exacerbation of Aβ plaque spread^37^ and tau protein abnormalities^38^. Similarly, TLRs, a component of the innate immune system, have also been implicated in AD. For example, misfolded Aβ can bind to TLRs to trigger the activation of the NF-kappa B pathway and subsequent release of pro-inflammatory cytokines^39^. Additionally, other studies have demonstrated the increased expression of TLR2, TLR4, TLR5, TLR7, TLR9, and the co-receptor CD14 in microglial cells surrounding senile plaques in the brain tissues of patients with AD and AD mouse models^40–42^.

Glucolipid metabolism is known to be closely associated with AD pathogenesis. In this context, inhibiting the PI3K-Akt pathway leads to insulin resistance, which in turn can result in tau phosphorylation and the promotion of Aβ deposition^43^. Moreover, ApoE serves as the principal apolipoprotein and cholesterol transporter in the lipid metabolism process in the central nervous system, while also being implicated in the increased incidence and total number of Aβ fibers^30^. Furthermore, Aβ aggregation is reported to occur due to the overproduction of Aβ and/or its inefficient clearance. Additionally, the ApoE genotype has been demonstrated to regulate the progression of amyloid pathology in the brain, with different human ApoE subtypes primarily affecting Aβ clearance and aggregation and ultimately influencing AD pathogenesis^44^. All these findings correspond well with the enrichment results obtained in our gene intersection analysis, indicating the presence of genes within the set of intersected genes that are pivotal in AD occurrence and progression. Overall, these collective studies provide supporting evidence for the relevance of these intersecting genes in AD, highlighting their critical involvement in the pathogenesis and development of this debilitating disease.

We also conducted LASSO and random forest machine learning analyses to screen the intersection genes and detected four hub genes: P2RX7, HSP90AA1, NT5E, and NLRP3. The expression levels of these central genes were significantly different between the healthy control and AD brain tissues. Specifically, HSP90AA1 and P2RX7 were significantly upregulated in the AD brain tissues, whereas NT5E and NLRP3 exhibited significantly lower expression levels. ROC curve analysis was further performed to evaluate the diagnostic potential of these hub genes in AD. All four genes demonstrated AUC values > 0.7, indicating their diagnostic value for AD. Furthermore, we investigated the protein levels of these four genes in the hippocampus of 3xTg-AD and C57BL/6J (background control) mice. Our results found that the expression levels of P2RX7 and HSP90AA1 were consistent with the trend observed in the brain tissue analysis.

P2RX7 is widely distributed in the central nervous system and expressed in neurons, astrocytes, and microglia^45–47^. Microglia are essential components in neuroinflammation, and the increased expression of microglial P2RX7 has been observed in humans and rodents exposed to Aβ^48^. Moreover, research on the AD brain has shown that activating microglial P2RX7 releases pro-inflammatory cytokines, while its inhibition alleviates neuroinflammatory responses^49^. In vitro studies have also demonstrated that Aβ promotes ATP and IL-1β release from primary microglia, whereas P2RX7 knockout mice do not exhibit this Aβ-induced IL-1β accumulation, suggesting that microglial P2RX7s are vital regulators of the AD inflammatory response^50^. Additionally, direct hippocampal injection of Aβ_1–42_ in a rat model resulted in P2RX7 upregulation in the hippocampal microglial cells, along with memory deficits and hippocampal neuron degeneration^48,51^. Another study revealed that treatment with brilliant blue G, a selective P2RX7 antagonist, conferred hippocampal neuroprotection, reduced inflammatory responses, and attenuated reactive gliosis^52^.

HSP90AA1 (HSP90α) belongs to the HSP90 family and is pivotal in maintaining the stability and functionality of numerous “client proteins” (including tau proteins) involved in cell survival, metabolism, and growth^53^. For example, HSP90 has been shown to indirectly regulate tau protein phosphorylation via tau kinases^54,55^, while inhibiting HSP90 decreases tau protein phosphorylation levels^56^. Therefore, targeting HSP90 may be a potential strategy to reduce tau kinase activity and mitigate AD progression^57^. Apart from its involvement in tau pathology, HSP90 is also associated with protection against Aβ toxicity. In this process, extracellular HSP90 enhances the phagocytosis and degradation of Aβ by microglial cells via the activation of the TLR4 receptor pathway, thereby promoting Aβ clearance^50,58^. However, HSP90 may exert a dual effect. Excessive HSP90 has been suggested to cause microglial hyperactivation, releasing pro-inflammatory cytokines and triggering a cascade of inflammatory reactions that ultimately lead to neuronal damage^59^. Moreover, other studies have demonstrated that administering HSP90 inhibitors in primary neurons can prevent Aβ-induced neurotoxicity^60^.

## Conclusion

Our study findings indicate that the ICD-mediated increase in the expression levels of HSP90AA1 and P2RX7 may play a prominent role in AD pathogenesis. Additionally, the elevated HSP90AA1 and P2RX7 levels could in turn lead to tau hyperphosphorylation and neuroinflammation, two critical contributors to this neurodegenerative disease. Overall, these results suggest that targeting the mechanisms involved in ICD and its associated molecular pathways, such as HSP90AA1 and P2RX7, may be a promising approach to developing novel therapeutic strategies for AD.

### Supplementary Information

Supplementary Information.

Supplementary Table S1.

Supplementary Figure 1.

## Data Availability

The datasets generated in this current study are publicly available.

## Abbreviations

AD: Alzheimer’s disease
ICD: Immunogenic cell death
DEGs: Differentially expressed genes
GO: Gene ontology
KEGG: Kyoto Encyclopedia of Genes and Genomes
LASSO: Least absolute shrinkage and selection operator
ssGSEA: Single-sample gene set enrichment analysis
Aβ: Amyloid-beta
DAMPs: Damage-associated molecular patterns
ATP: Adenosine triphosphate
HSPs: Heat shock proteins
HMGB1: High mobility group box 1
APCs: Antigen-presenting cells
PRRs: Pattern recognition receptors
GEO: Gene Expression Omnibus
WGCNA: Weighted gene co-expression network analysis
ROC: Receiver operating characteristic
BP: Biological process
CC: Cellular component
MF: Molecular function
CI: Confidence interval
AUC: Area under the curve
TLR: Toll-like receptor
