# Supplementary material for: Identification of immunogenic cell death-related genes involved in Alzheimer’s disease
Source: Sci Rep. 2024 Feb 15;14:3786. doi: 10.1038/s41598-024-54357-6 (PMC10869701; doi:10.1038/s41598-024-54357-6)
Supplement: Supplementary file 1 — Supplementary Information. [file 41598_2024_54357_MOESM1_ESM.docx]

**R package**

| Name | Version |
| --- | --- |
| pheatmap | 1.0.12 |
| venn | 1.11 |
| sva | 3.40.0 |
| glmnet | 4.1.2 |
| ggpubr | 0.4.0 |
| GSVA | 1.40.1 |
| vioplot | 0.4.0 |
| limma | 3.48.3 |
| WGCNA | 1.72.1 |
| tidyverse | 1.3.1 |
| reshape2 | 1.4.4 |
| org.Hs.eg.db | 3.16.0 |
| clusterProfiler | 4.0.5 |
| enrichplot | 1.12.2 |
| pROC | 1.18.0 |
| ggplot2 | 3.3.5 |
| dplyr | 1.1.2 |
| ggrepel | 0.9.1 |
