# Supplementary material for: Identification of immunogenic cell death-related genes involved in Alzheimer’s disease
Source: Sci Rep. 2024 Feb 15;14:3786. doi: 10.1038/s41598-024-54357-6 (PMC10869701; doi:10.1038/s41598-024-54357-6)
Supplement: Supplementary file 2 — Supplementary Table S1. [file 41598_2024_54357_MOESM2_ESM.docx]

Table S1 Immunogenic cell death related genes

| Immunogenic cell death related genes |
| --- |
| ATG5 |
| ADA |
| AKT1 |
| ATM |
| AXL |
| BAX |
| BCL2 |
| BRAF |
| CALR |
| CASP1 |
| CASP8 |
| CD4^+^ |
| CD8^+^A |
| CD8^+^B |
| CDKN2A |
| CENPA |
| CRT |
| CTNNB1 |
| CXCR3 |
| DKK3 |
| EGFR |
| EIF2AK3 |
| ENTPD1 |
| FAS |
| FOXP3 |
| HMGB1 |
| HRAS |
| HSP90AA1 |
| IFNA1 |
| IFNB1 |
| IFNG |
| IFNGR1 |
| IL10 |
| IL17A |
| IL17RA |
| IL1B |
| IL1R1 |
| IL5RA |
| IL6 |
| LY96 |
| MYCN |
| MYD88 |
| NLRP3 |
| NT5E |
| P2RX7 |
| PDIA3 |
| PIK3CA |
| PRF1 |
| PTCH1 |
| PTEN |
| SCN5A |
| SLAMF7 |
| TIMP1 |
| TLR4 |
| TNF |
| TP53 |
| TREM1 |
