# Supplementary material for: Identification of immunogenic cell death-related genes involved in Alzheimer’s disease
Source: Sci Rep. 2024 Feb 15;14:3786. doi: 10.1038/s41598-024-54357-6 (PMC10869701; doi:10.1038/s41598-024-54357-6)
Supplement: Supplementary file 3 — Supplementary Figure 1. [file 41598_2024_54357_MOESM3_ESM.pdf]

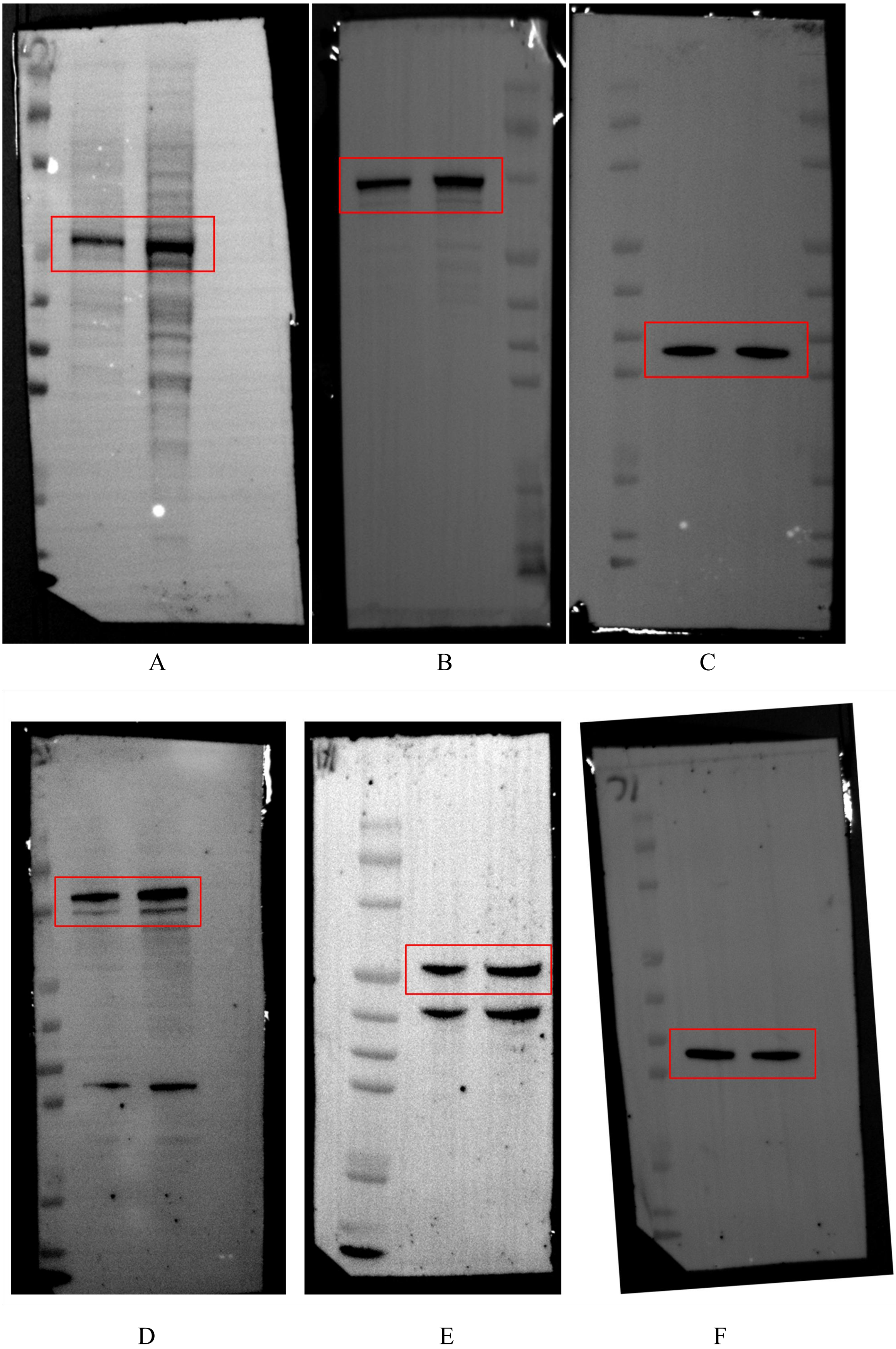

Supplementary Figure1:Original western blot images

A(P2RX7), B(HSP90AA1), C(GADPH), D(NLRP3), E(NT5E), F(GADPH)
